# Chemiluminescent carbon nanodots for dynamic and guided antibacteria

**DOI:** 10.1038/s41377-023-01149-8

**Published:** 2023-05-04

**Authors:** Jiang-Fan Han, Qing Lou, Zhong-Zheng Ding, Guang-Song Zheng, Qing-Chao Ni, Run-Wei Song, Kai-Kai Liu, Jin-Hao Zang, Lin Dong, Cheng-Long Shen, Chong-Xin Shan

**Affiliations:** Henan Key Laboratory of Diamond Optoelectronic Materials and Devices, Key Laboratory of Material Physics, Ministry of Education, School of Physics and Microelectronics, Zhengzhou University, Zhengzhou, 450052 China

**Keywords:** Biomaterials, Nanoparticles

## Abstract

Advanced antibacterial technologies are needed to counter the rapid emergence of drug-resistant bacteria. Image-guided therapy is one of the most promising strategies for efficiently and accurately curing bacterial infections. Herein, a chemiluminescence (CL)-dynamic/guided antibacteria (CDGA) with multiple reactive oxygen species (ROS) generation capacity and chemiexcited near-infrared emission has been designed for the precise theranostics of bacterial infection by employing near-infrared emissive carbon nanodots (CDs) and peroxalate as CL fuels. Mechanistically, hydrogen peroxide generated in the bacterial microenvironment can trigger the chemically initiated electron exchange between CDs and energy-riched intermediate originated from the oxidized peroxalate, enabling bacterial induced inflammation imaging. Meanwhile, type I/II photochemical ROS production and type III ultrafast charge transfer from CDs under the self-illumination can inhibit the bacteria proliferation efficiently. The potential clinical utility of CDGA is further demonstrated in bacteria infected mice trauma model. The self-illuminating CDGA exhibits an excellent in vivo imaging quality in early detecting wound infections and internal inflammation caused by bacteria, and further are proven as efficient broad-spectrum antibacterial nanomedicines without drug-resistance, whose sterilizing rate is up to 99.99%.

## Abstract (web summary)

Chemiluminescence-dynamic/guided antibacterial agents with multiple reactive oxygen species generation capacity and chemi-excited near-infrared emission from carbon nanodots have been designed for precise theranostics of bacterial infection.

## Graphical abstract

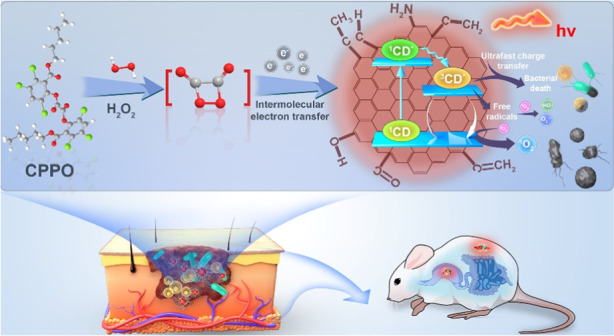

## Introduction

Antimicrobial resistance poses huge threats to global public health, thus developing effective antimicrobial agents and antimicrobial technologies are crucial for reducing bacterial infections and preventing bacterial resistance. Photodynamic therapy (PDT) through the photosensitizers excited by light, which employs the produced reactive oxygen species (ROS) to kill pathogenic bacteria, has emerged as a clinical therapy to various bacteria-related infections^1,2^. Compared with the traditional antibiotic therapies, the PDT-based antibiosis possesses unique advantages of noninvasiveness, broad spectrum, high temporal and spatial resolution and the absence of drug-resistance, etc.^3^. However, the requirement of external light source for the PDT has seriously limited its application in the treatment of deep tissue lesions^4–7^. Chemiluminescence (CL), as one kind of luminous emission triggered by chemical reaction, has evoked widespread attention in diverse applications like immunodetection, cold light illuminator and bioimaging^8,9^. Driven by the CL derived from the enriched hydrogen peroxide (H_2_O_2_) in tumor microenvironment and energetic intermediates, the in situ generated ROS can achieve efficient tumor cell killing without the external light excitation, which has been proved with its splendid oncology therapy ability^10–13^. Hence, with the CL emission by the interaction between the CL substrate and excessive H_2_O_2_ caused by the bacteria-related inflammation or self-release, CL-based PDT system can be further employed to achieve in vivo or in vitro diagnosis and therapy of bacteria-related infections^13^. Nevertheless, a good CL-based PDT system generally requires a suitable photosensitizer with both deeply-tissue-penetrating long-wavelength CL emission and efficient ROS production capacity^14–17^. To date, most of the currently available photosensitizers are limited by their short emission wavelength, low CL quantum yield (CL QY) or inadequate productivity of ROS^18–20^. In addition, extra H_2_O_2_ or coelenterazine reagents used in previously reported self-luminescent systems may inevitably trigger normal tissue injury. Moreover, their low bactericidal effectiveness makes it challenging to treat infectious diseases clinically. Thus, it is still an urgent requirement to develop new class of CL fluorophore and therapeutic strategy^21^.

Carbon nanodots (CDs), which are defined as a group of small sized carbon nanostructures with a diameter below 10 nm, have been investigated extensively since their discovery in 2004^22–25^. For decades, CDs have attracted great attention due to their unique advantages such as tunable emission wavelength, high photoluminescence quantum yield (PL QY), low-cost raw materials, excellent biocompatibility, low cytotoxicity, etc.^26,27^. Recently, CDs have been proved as one efficient fluorophore with CL emission in peroxalate-H_2_O_2_ system^28^. Meanwhile, CDs has been confirmed the capability of ROS generation due to their unique carbon based sp^2^ hybrid structure, endowing their promising applications in PDT for oncology and antibacterial activity^1,29^. Most recently, we have developed one kind of CD-based CL nanogels and further achieved effective oncotherapy, demonstrating their potential of in situ cytotoxic ROS production to kill cancer cells^30^. Therefore, it is highly promising to design a simple CD-based CL system to realize bright long-wavelength emission, high-efficiency ROS generation and novel dynamic therapy for the treatment of in-depth bacterial infection or inflammation. Nevertheless, none such report can be found up to date.

In this work, one kind of CL-dynamic/guided antibacteria (CDGA) using a photosensitive near-infrared (NIR) emissive CDs has been designed. For the formation of the CDGA, the CDs are employed as monomers of a self-illuminating platform to self-assemble with the amphiphilic polymeric conjugate (Pluronic 127, F-127) and CL donors (bis(2,4,5-trichloro-6-carbopentoxyphenyl) peroxalate, CPPO), precipitating to construct H_2_O_2_-driven water-soluble CDGA with the emission centered at 675 nm. Their corresponding CL QY is up to 1.93 × 10^−4^ einsteins mol^−1^, comparable with the values ever reported NIR CL fluorophores. Mechanistically, H_2_O_2_ generated in the bacteria-related inflammatory sites and self-release can trigger the chemically initiated electron exchange luminescence (CIEEL) between the CDs and intermediate originated from the redox reaction of peroxalate, enabling in vitro and in vivo CL imaging for ROS monitoring. Meanwhile, the production of type I/II photochemical ROS such as singlet oxygen anion (^1^O_2_), hydroxyl radicals (•OH) and superoxide anion (•O_2_^−^) can efficiently inhibit the bacteria growth and the novel type III ultrafast excited electron transfer from CDs to RNA exists in the system may inhibit the bacteria proliferation efficiently^31,32^. Finally, the self-illuminating CDGA exhibits an excellent in vivo imaging quality in detecting wound infections and internal inflammation caused by bacteria toxin at early stage, and it presents a 96.94–99.99% bacteriostatic rate toward gram-positive/negative bacteria, outperforming other CL fluorophores. The wound infection induced by gram-positive and gram-negative bacteria in model mice can be healed better than commercial adhesive bandage through a 14-day treatment with CDGA, demonstrating its potential clinical utility. Thus, the CL CDGA are expected to be as nanomedicines to kill broad-spectrum and drug-resistance bacteria efficiently and evaluate the early bacterial infection simultaneously under a desirable safety profile.

## Results

**Fig. 1:**
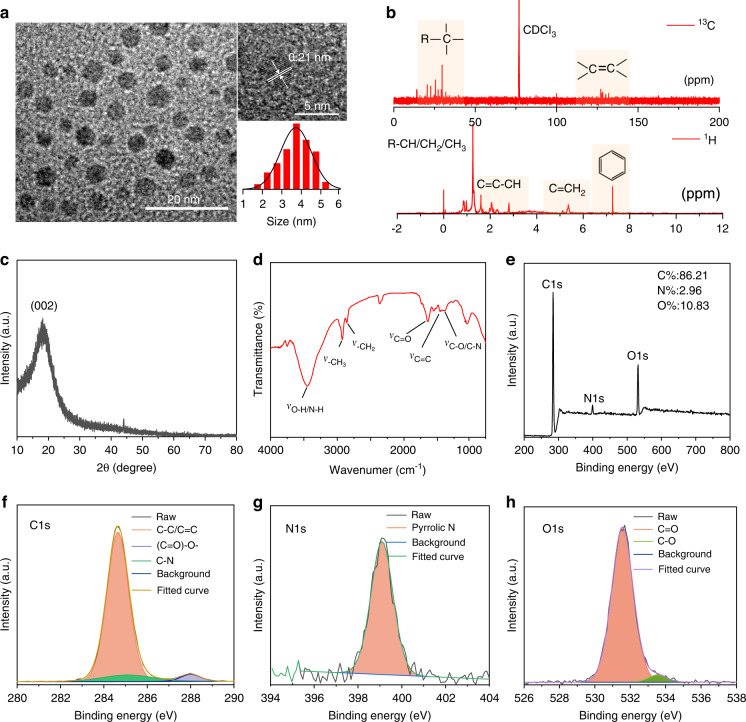
Characterization of the CDs. **a** Transmission electron microscopy (TEM) image, high-resolution TEM (HRTEM) image and the size distribution of the CDs. **b** The ^1^H and ^13^C NMR spectra of the CDs in CDCl_3_. **c** The XRD pattern of the CDs. **d** Fourier transform infrared (FT-IR) spectrum of the CDs. **e** Full survey XPS of the CDs. The high-resolution XPS spectra of the C 1s (**f**), N 1s (**g**), and O 1s (**h**) for the CDs

The NIR emissive CDs are obtained from the platanus leaves via a solvothermal strategy (Fig. S1). The contrast tests have been performed with different heat treatment or raw material conditions and the obtained optimal products are investigated. As presented in Fig. S2, the products obtained from 60 to 220 °C illustrate different PL emission as the CDs and the products obtained at 140 °C illustrate the highest deep-red emission with low peak around. Meanwhile, the products obtained in DMF, THF, DMSO and H_2_O illustrate similar PL emission as the CDs prepared in EtOH, which illustrate the highest PL intensity. As a result, the experiment condition used in the work is the best parameter for the preparation of CDs. On the condition, the productivity (Q) of the CDs, which is defined as the mass ratio of the as-prepared CDs and total mass of precursor, is calculated as high as 12% (Fig. S3). In addition, the products obtained from different plants exhibit different luminescent characteristics from the CDs. And the possible reason is deduced that the plant-derived CDs are formed by the carbonization and polymerization of the related molecules derived from these varied plants, the difference of initial raw materials may largely lead to the difference of luminescence properties due to the otherness of particle size, doping elements and surface groups in final products (Fig. S4). As shown in Fig. 1a, the morphologies of the CDs are investigated with the transmission electron microscopy (TEM) by depositing their solution on copper grids. In the TEM image, the CDs present a spherical shape with an average diameter of around 3.7 nm and the high-resolution TEM (HRTEM) image reveals that the CDs possess well-resolved lattice spacing of 0.21 nm, corresponding to the (100) plane of graphitic carbon^33^. As shown in Fig. S5, the heights of the CDs are around 6 nm in atomic force microscopy (AFM) images, which are corresponding to the size distribution in TEM image. As exhibited in Fig. S6, the hydration sizes of the CDs in aqueous solution are further investigated with the dynamic light scattering (DLS) and the result presents the size distributions around 459.1 nm. The different size distribution of the CDs in DLS and TEM image is deduced from the polymer chains on the surface of CDs, which can promote the further polymerization of carbon nanodots into nano-micelles through hydrogen bond interaction in water^30,34^. With the nuclear magnetic resonance (NMR) spectroscopy and X-ray diffraction (XRD), the structure and chemical composition of the CDs are further investigated. As shown in Fig. 1b, the ^13^C NMR spectrum illustrate the signals in the range of 0–40 ppm ascribed to the sp^3^ C atoms and the signals in the range of 110–140 ppm range attributed to the sp^2^ conjugated aromatic carbon atoms. In the ^1^H NMR spectrum, the H signals in the range of 0–2.5 ppm attributed to the –C-H/-C-CH_2_/-C-CH_3_ and 5.0–6.0 ppm attributed to the C=CH_2_ can be observed. Especially, the active H signals in the range of 6 to 8 ppm confirm the existence of aromatic hydrogen^34^. Meanwhile, the XRD pattern of the CDs shows a main peak at around 23°, which can be attributed to the (002) planes of graphitic carbon (Fig. 1c), implying the high crystalline of the CDs^35,36^. In addition, the functional groups of the CDs are further characterized by Fourier transform infrared (FT-IR) and X-ray photoelectron spectroscopy (XPS). As shown in Fig. 1d, the FT-IR spectrum of the CDs reveals the peak of 3450 cm^−1^, which can be attributed to the vibration of O–H and N–H. The peaks at around 2930 and 2850 cm^−1^ can be attributed to the vibration of –CH_3_ and –CH_2_, respectively. And, the peak at around 1650, 1460 and 1400 cm^−1^ can be ascribed to C=O, C=C and C–O/C–N, respectively^37,38^. Meanwhile, the XPS survey spectrum confirms the composition of C (284.6 eV), N (399.6 eV), and O (532.6 eV) elements in the CDs (Fig. 1e), which is consistent with the FT-IR analysis. In the high-resolution XPS spectra, the C1s envelope can be deconvoluted into C=C/C–C (284.6 eV), C–N (285.1 eV), and (C=O)-O- (288.0 eV) (Fig. 1f), the N1s envelope can be fitted with pyrrolic N (399.1 eV) (Fig. 1g), and the O1s envelope can be C=O (531.5 eV) and C–O (533.5 eV) (Fig. 1h)^39,40^. Hence, the obtained CDs are consisted of sp^2^ conjugated aromatic structure with abundant N/O groups grafted on their surface, which may facilitate the electron transfer in their CL system.

**Fig. 2:**
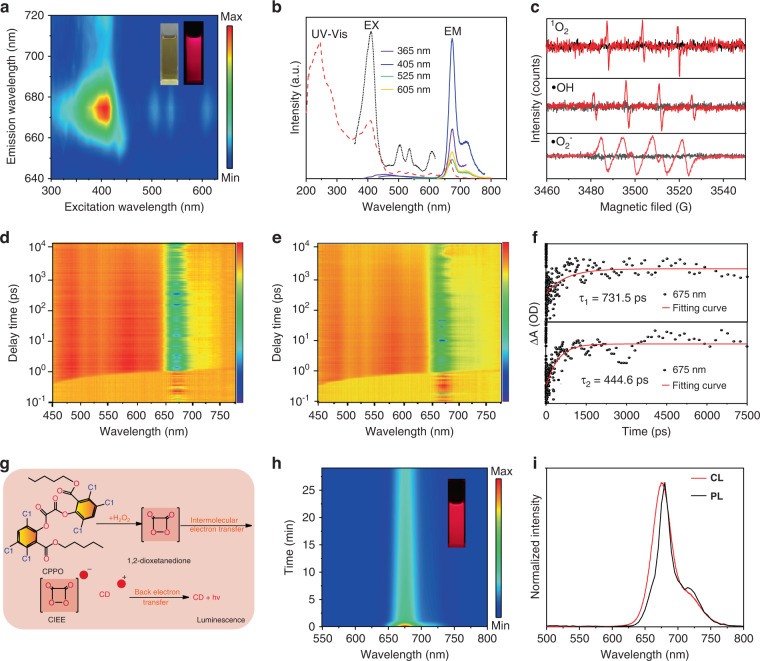
The photophysical properties of the CDs. **a** The excitation-emission matrix of the CDs ethanol solution (insets: the photograph of the CDs solution under sunlight and 365 nm UV lamp). **b** The UV–vis absorption spectra, PL excitation (EX) and emission (EM) spectra of the CDs ethanol solution. **c** The ESR spectrum upon light irradiation of the CDs for detecting the ROS species of ^1^O_2_, •OH and •O_2_^−^ (Red line: Light conditions, Black line: Dark conditions). 3D pseudo-color map (in bird’s eye) of transient absorption (TA) spectra of CDs (**d**) and CDs mixed with bacteria (**e**) and their corresponding fitting curves (**f**). **g** Schematic illustration of the CL process induced by the CIEEL. **h** The time-dependent CL spectra after adding the CDs into the mixture of CPPO and H_2_O_2_ (inset: the photograph of the CL solution). **i** The normalized CL and PL emission spectra of the CDs

The photophysical properties of the CDs are further investigated. As shown in Fig. S7a, the PL intensity of the CDs can keep invariant after irradiated continuously with a 365 nm UV lamp for 30 min. Meanwhile, the CDs illustrate no any obvious degradation in the PL intensity when exposed to natural light for 2.5 h (Fig. S7b). As a result, the chemiluminescent CDs have good photostability in natural or ultraviolet environment. As shown in Fig. 2a, bright red fluorescence can be observed from the CDs ethanol solution under 365 nm UV lamp by naked eyes, as similar as the CDs solution dispersed in DMSO, DMF and THF (Fig. S8a–c). However, the CDs in aqueous solution exhibit far lower fluorescence intensity than these CDs in organic solution (Fig. S8d). Meanwhile, the PL of these CDs in water/ethanol mixtures with different water contents become weakened and the PL lifetimes of these CDs solution decrease with the increased water content (Fig. S9), confirming the formative CDs aggregation triggered by high polar water and the corresponding aggregation-caused quenching (ACQ) behavior^41^. In addition, the 3D excitation-emission matrix of the CDs ethanol solution exhibits an excitation-emission center at (410 nm, 675 nm). Under the different excitation wavelength ranging from 300 to 650 nm, the CDs in ethanol solution illustrate the similar PL peak around 675 nm, implying the excitation-independent emission (Figs. 2b and S10). In addition, the CDs ethanol solution presents two absorption bands at 415 and 675 nm in the UV–vis absorption (Figs. 2b and S11), which are attributed to the transition n → π* and π → π*. Since the CDs solution exhibit excellent PL emission, different contents of ROS can be produced by the CDs in aqueous solution under light irradiation. As shown in Fig. 2c, the productions of dissolved oxygen derived type II ^1^O_2_ and H_2_O derived type I •OH/•O_2_^−^ can be detected by electron paramagnetic resonance (ESR) using DMPO (5,5-dimethyl-1-pyrroline N-oxide), TEMPO (2,2,6,6-tetramethylpiperidine-1-oxyl) and TEMP (2,2,6,6-tetramethyl-1-piperidine) as spin probes^42^. As previous reports^32^, the photosensitizers can bind to RNA without interference by other species and transfer excitation-state energy directly to destroy RNA in cancer cells without the dependence of oxygen (type-III mechanism). To gain further insight into the electron transfer dynamics between CDs and bacteria, the femtosecond transient absorption (fs-TA) of CDs solution at 675 nm under free condition and CDs mixed with bacteria were measured (Fig. 2d, e). Herein, the fitted curve exhibited the lifetime of 731.5 ps for CDs and 444.6 ps for CDs mixed with bacteria, in which the shorter lifetime of CDs mixed with bacteria indicated there is an electron exchange process between CDs and bacteria (Fig. 2f). Hence, the excited CDs can be also bound to the biological macromolecules of RNA from bacteria, and the novel type III ultrafast excited electrons transferred from CDs to RNA exist in the bactericidal system may inhibit the bacteria proliferation efficiently. Moreover, with the CIEEL, the CDs can emit bright NIR CL emission in the CL system of peroxalate and H_2_O_2_ (Fig. 2g). In the process, the spontaneous oxidation reaction of peroxalate compound (CPPO) and H_2_O_2_ can yield energetic intermediates of 1,2-dioxetanedione, and then the intermolecular electron exchange between the emitter and the intermediate can lead to the excitation of CDs, resulting in the CL emission of CDs. As shown in Fig. 2h, the bright deep-red CL can be observed by naked eyes after adding the CDs into the mixture of CPPO and H_2_O_2_. With the F-7000 spectrofluorometer under lamp off, the light emission spectrum of the CL emission can be measured, which exhibits one narrow peak around 675 nm as similar as their PL emission (Fig. 2i). With the lucigenin-H_2_O_2_ reaction as reference, the corresponding CL QY of the CDs can be calculated as 1.93 × 10^−4^ einsteins mol^−1^ (Fig. S12), which are comparable with the values in ever reported NIR CL fluorophore^28,33,43^.

**Fig. 3:**
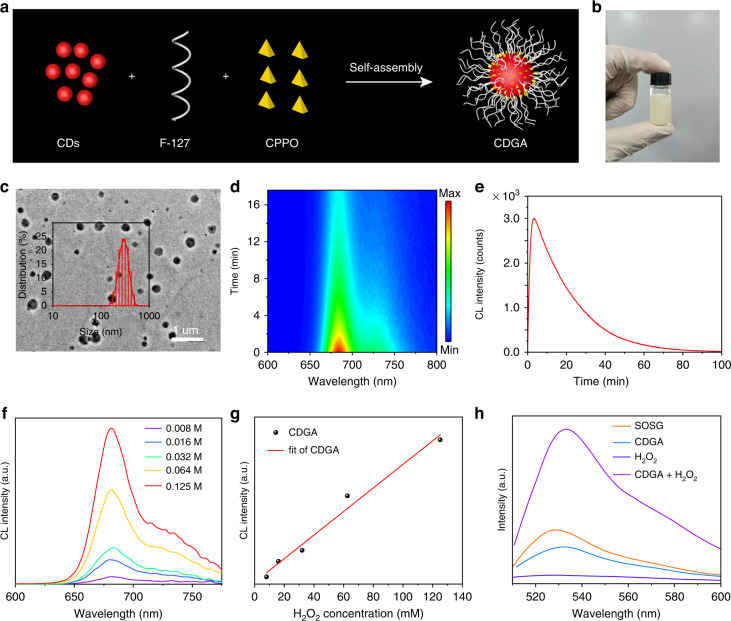
Synthesis and characterization of the CDGA. **a** Schematic illustration of the preparation of the CDGA. **b** The photograph of the CDGA aqueous solution under sunlight. **c** The TEM image of the CDGA (inset: the size distribution of the CDGA in DLS). **d** The time-dependent CL spectra after adding the CDGA into H_2_O_2_ aqueous solution. **e** The CL decay curve after adding the CDGA into H_2_O_2_ aqueous solution. **f** The CL spectra of the CDGA under different concentrations of H_2_O_2_. **g** The integral CL intensity of CDGA vs. the concentrations of H_2_O_2_. **h** Determination of the production of ^1^O_2_ under dark condition in aqueous solutions of the CDGA

With the excellent CL emission and ROS generation capability, the CDs are employed as one kind of chemiexcited photosensitive emitter to design CDGA. As shown in Fig. 3a, the CDs are employed as emitters of a self-illuminating platform to self-assemble with the amphiphilic polymeric conjugate (F-127) and CL donors (CPPO), precipitating the H_2_O_2_-driven CDGA. The obtained CDGA exhibit excellent water-solubility (Fig. 3b), implying their promising applications in diverse biomedicines. The TEM image confirms that the CDGA possess poly-dispersed morphology with a size distribution ranging from 50 to 500 nm, which is corresponding to the investigation from the DLS (Figs. 3c and S13). We have recorded the characters of the antibacterial colloid film. As shown in Fig. S14a, b, the CDGA film exhibited the fibrous textures with porous surface from the scanning electron microscope (SEM) image and machinable and uniform film characteristics can be observed from the optical photograph. Meanwhile, the CDGA aqueous solution exhibit similar PL excitation and emission spectra as the initial CDs solution (Fig. S15a). Moreover, the CDGA aqueous solution present the similar UV–vis absorption spectra as the CDs while there are no absorption bands at 415 and 675 nm for the F-127 and F-127 + CPPO, indicating that the fluorescence of CDGA are originated from the CDs (Fig. S15b). Similarly, the CDGA solution can emit the same NIR CL emission as the pristine CDs after adding H_2_O_2_ aqueous solution (Fig. 3d) and the corresponding CL spectra are similar as their PL emission (Fig. S16). The CL emission of the CDGA can persist over 60 min, which is beneficial to the long-term bioimaging (Fig. 3e). The selectivity of the CL from CDGA for the detection of H_2_O_2_ is further studied with CL analysis instrument. As presented in Fig. S17, the CL signal of the CDs in the presence of H_2_O_2_ is far higher (>100 times) than that in the presence of other ROS (OCl^–^, ONOO^–^, •OH, ^1^O_2_), confirming the high selectivity of CDGA toward H_2_O_2_. With the direct measurement of F-7000 spectrofluorometer, the CL emission after adding the CDGA into the H_2_O_2_ aqueous solution exhibit excellent linear relationship between the CL intensity and the concentration of H_2_O_2_ (Fig. 3f, g), confirming the promising application of CDGA in H_2_O_2_ sensing. Moreover, the CDGA exhibit excellent ^1^O_2_ generation capability after added into H_2_O_2_ aqueous solution. As shown in Fig. 3h, the CDGA exhibit the obvious ^1^O_2_ signals after adding CDGA to H_2_O_2_ aqueous solution under dark conditions when the probes of singlet oxygen sensor green (SOSG) are used to test the ^1^O_2_^44^. Compared with the single SOSG, CDGA or H_2_O_2_ aqueous solution, the CDGA + H_2_O_2_ + SOSG aqueous solution illustrate obvious higher green fluorescence, confirming the CL-initiated photodynamic capability of CDs.

**Fig. 4:**
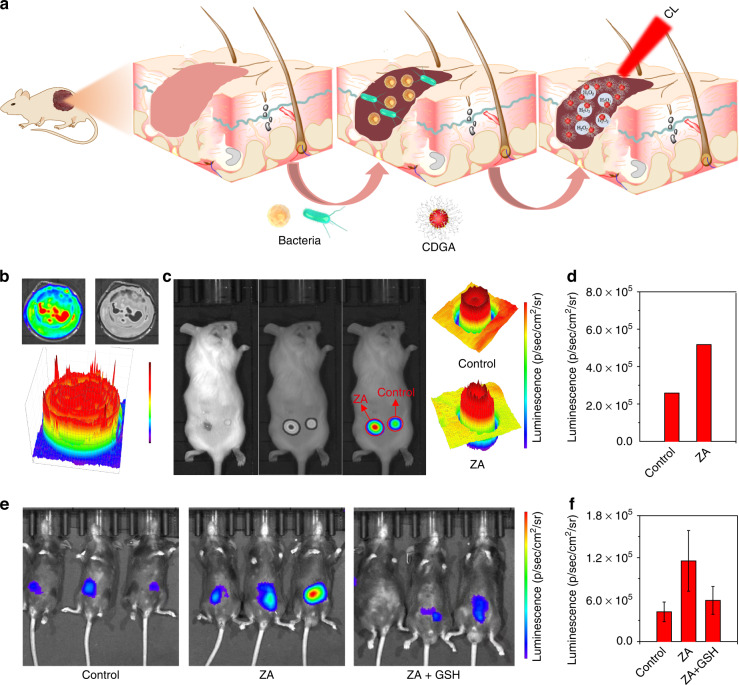
In vitro and in vivo bioimaging of bacteria-associated inflammation. **a** Schematic illustration of the CL sensing in inflammation model. **b** The CL emission of the CDGA recorded by the IVIS system. **c** The CL images of mice treated with and without the ZA in superficial wound. **d** The quantification of corresponding CL intensity from the images of superficial wound. **e** The CL images of mice intraperitoneally treated with ZA, ZA plus GSH and saline, following by an intraperitoneal injection of CDGA at *t* = 4 h. **f** The quantification of corresponding CL intensity from the in vivo CL images

With the merit of long persistent afterglow and deep penetration of the NIR emission, the CDGA can be employed as activatable imaging agents for in vivo CL image of inflammation-related ROS. As shown in Fig. 4a, the bacterial infection in the wound or deep tissue of living body can trigger the inflammation response, leading to the increased ROS levels^45^. Hence, the CDGA can be used for the CL probes to achieve the early diagnostics of the bacterial inflammation by monitoring the concentration of endogenous H_2_O_2_. As shown in Figs. 4b and S18, the mixed aqueous solution of CDGA and H_2_O_2_ illustrates diverse CL emission intensity in a laboratory dish, enabling the CDGA to describe the distribution of H_2_O_2_. Whereas, with the NIR CL emission and CL plot from the CL distribution, the in vivo CL imaging can be used to investigate the ROS level and distribution in different inflammation sites. Herein, two kinds of bacterial inflammation models are established with the bacterial exotoxin of ZA to induce inflammation and further generate excessive H_2_O_2_. As shown in Figs. 4c and S19, the epidermal wound models are established by directly treating the mouse wound with ZA to simulate the wound bacterial infection. In the model, the skin with wound can emit more intense CL emission compared with the common skin after spraying the CDGA aqueous solution (Fig. 4d), confirming the existence of ZA-induced inflammation. Meanwhile, the CL emission distribution in the wound can present the different severity of symptoms for the possible bacterial infection. Moreover, the CDGA also exhibit excellent performance in the in vivo CL bioimaging of deep-penetrated inflammation. The inflammatory mouse models are established through intraperitoneal injection with ZA to simulate the intra-abdominal bacterial infection. After 24 h, the deep-penetrated inflammation bioimages of mouse models are subsequently captured after the injection of the CDGA. As shown in Fig. 4e, the intra-abdominal sites of mice treated with ZA exhibit higher CL signals compared with the control group. The CL diagnostic signals of ZA-treated mice with deep abdominal tissue are almost 2.5-times higher than that for the control mice. Moreover, after the infected mice with deep abdominal tissue are remedied with an antioxidant glutathione (GSH), the CL signals of the ZA/GSH-treated mice show a 40% reduction (Fig. 4f). As an inflamed-to-normal contrast signals, the enhanced-to-reduced CL intensity can efficiently monitor the variation of bacterial infection in living animals. To evaluate the advantage of CL in the tissue penetration, the tissue penetration depth of the CD-based CL and FL has been compared. As shown in Fig. S20a, the CL signals can be still detected when the tissue depth are more than 1 mm, while the FL signals cannot be acquired when the tissue depth are more than 1 mm. The valid tissue penetration depth of CL image can be calculated 2.3 mm as while the FL image is 0.01 mm (Fig. S20b), indicating the better performance of bioimaging ability for CL than FL. The stability of CDGA is important for CL imaging. As shown in Fig. S21, the CL intensity of the CDGA film at 675 nm changes slightly after stored for 5 days, indicating the excellent CL stability of CDGA film. Finally, we have also tested the influence of the thickness and exposure time of the CDGA film for the CL image. As displayed in Fig. S22a, c, the CDGA films with different thickness exhibit similar CL intensity at 675 nm, implying the low influence of the CDGA thickness on the CL imaging. Meanwhile, the CDGA films under more exposure time exhibit higher CL intensity, indicating the importance of exposure time in the CL imaging (Fig. S22b, d). Although high exposure time will increase the image contrast, it will obviously increase the time spent in the experiment under actual operation. Therefore, the specific exposure time of CL biological imaging is based on the comprehensive consideration of image contrast and time economy. These investigations indicate the CDGA can be used as inflammation-responsive imaging agent in living body, which is potential for the long-term bioimaging and bacterial infection monitoring.

**Fig. 5:**
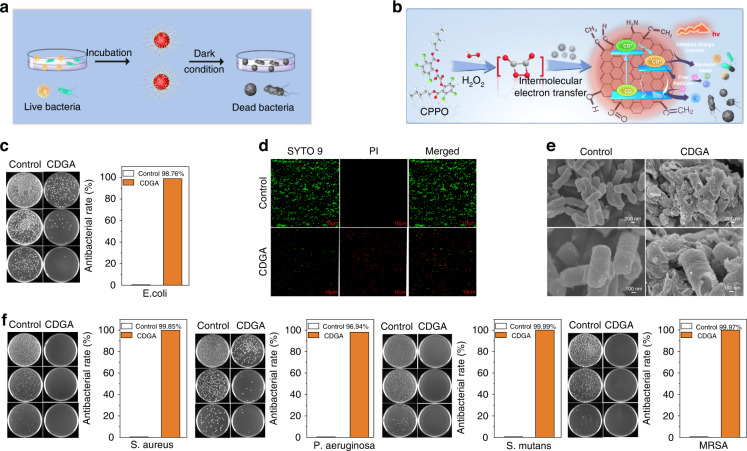
In vitro antibacterial activities of CDGA. **a** Schematic illustration of the broad-spectrum antibacterial measurement for the CDGA. **b** The antibacteria mechanism of the CDGA. **c** The flat counting images of E. coli treated with and without the CDGA without light irradiation. **d** The CLSM images of the bacteria stained by SYTO 9 and PI, respectively. Scale bar = 10 um. **e** The SEM images of E. coli treated with and without the CDGA without light irradiation. **f** The flat counting images of S. aureus, P. aeruginosa, S. mutans and MRSA treated with and without the CDGA without light irradiation and corresponding inactivation rates

As previous report, the production of ^1^O_2_, •OH and •O_2_^−^ from photochemical reaction can cause effective oxidative damage on the bacterial membrane^1,46^. In the sites of bacterial infection, the inflammatory microenvironment with highly expressed H_2_O_2_ can trigger the CL emission of CDGA and further achieve self-illuminated PDT, implying their potential applications as nanomedicine for the antibacterial applications. As shown in Fig. 5a, the antimicrobial properties of the CDGA are investigated. Mechanistically, the ROS generated in the bacteria-related inflammatory sites can trigger the CIEEL. The type III ultrafast charge transfer from CDs and the production of such as ^1^O_2_, •OH and •O_2_^−^ from the CDGA under the self-illumination can efficiently inhibit the bacteria growth (Fig. 5b)^47^. In this work, the Flat counting images of E. coli treated with the F-127, F-127 + CPPO, F-127 + CDs and CDGA under dark condition are firstly investigated. As shown in Figs. 5c and S23, the experiment with adding the CDs exhibit the bacteriostatic rate of 98.76% while the experiment without adding the CDs show a lower bacteriostatic rate of 74.67%, indicating the importance of the CDs in the antibiotic therapy. Meanwhile, pure CDs also play an ineffective role in sterilization, further confirming the production of type I/II photochemical ROS and type III ultrafast charge transfer from the CDGA can efficiently inhibit the bacteria growth. On the condition, the antibacterial activities of the CDGA intuitively are further investigated. As shown in Fig. 5d, e, the viabilities and morphologies of E. coli before and after treated with CDGA are observed by the LIVE/DEAD staining and SEM. In the confocal laser scanning microscope (CLSM) images of SYTO 9 and PI stained bacteria, the control group without CDGA treatment exhibit green fluorescence, corresponding to the normal living bacteria. However, the experimental group treated with the CDGA show bright red fluorescence, indicating the death of bacteria induced by the CDGA. Meanwhile, the SEM images exhibit obvious morphological changes. In these E. coli bacteria, the control group exhibit spherical shapes and rod-shaped, with glossy and unharmed membranes, while the bacteria incubated with CDGA display partial wrinkling and disruption of bacterial membrane. The altered membranes indicate the damages of bacterial membranes induced by the ROS-based PDT, which is consistent with the expected antibacteria mechanism of CDGA. As illustrated in Fig. S24a, b, with the increased concentration, the antibacterial rate of the CDGA increases with the increased concentration and the antibacterial rate reaches to the maximum when the concentration is up to 0.5 mg ml^−1^. Thus, the maximum concentration and dose can be set to this value of 0.5 mg ml^−1^ during the actual antibacterial use. Moreover, the CDGA exhibit broad-spectrum antibacterial capability. As shown in Fig. 5f, the CDGA exhibit the bacteriostatic rate of 99.85%, 96.94%, 99.99% and 99.97% for the S. aureus, P. aeruginosa, S. mutans and MRSA, respectively. These results confirm the promising applications of CDGA as one new class of broad-spectrum antibacterial medicines to resolve the bacterial drug resistance.

**Fig. 6:**
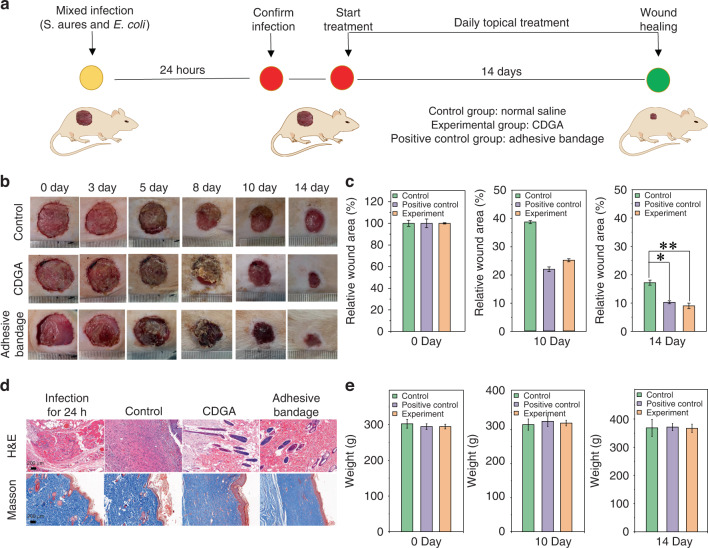
In vivo antibacterial capability of the CDGA. **a** The experimental process of mice trauma model with the mixed infection of S. aureus and E. coli. **b** The photographs of the infected wounds in negative control group (Control, normal saline), experimental group (CDGA), and positive control group (Adhesive bandage). **c** The relative wound area at 0, 10 and 14 days for different groups. (*p < 0.05, **p < 0.01). **d** The images of H&E staining and Masson’s staining, including infection for 24 h and treatment for 14 days. Scale bar = 200 μm. **e** The body weight variations at 0, 10 and 14 days for different groups

When the skin is compromised, the bacteria can infiltrate into the subcutaneous tissue that is physiologically optimum for colonization and growth, leading to the wound infection and further prevent wound healing^48^. Nowadays, the wound infection induced by bacteria are still one huge challenge for human health^49^. In practice, the S. aureus and E. coli are representative gram-positive and gram-negative bacteria which can directly result in wound infection. The biosafety of the triblock copolymer polyether F-127 has been approved by the US Food and Drug Administration, which can act as FDA approved drugs for pharmaceutical and biomedical applications. With the central hydrophobic poly(propylene oxide) (PPO) block and two hydrophilic poly(ethylene oxide) (PEO) block, the polyether F-127 can cross-link the CDs to form nanogels due to the hydrophobic polymer chain of CDs. In addition, the CDGA can closely cover the wound and easily washed away by saline without secondary injury, indicating the promising applications of CDGA as flushable bandage (Fig. S25). Herein, the wound infection models are established with the composites of S. aureus and E. coli to evaluate the in vivo antibacterial effect of CDGA. As schematic illustration in Fig. 6a, the wound dressings of the control group, experimental group, and the positive control group are recorded. As illustrated in Fig. 6b, the infected skin wounds of the experimental group and the positive control group exhibit obvious scabs and less exudate than the control group. After 14 days, the wound areas of the experimental group and the positive control group are significantly smaller (Fig. 6c). Meanwhile, the corresponding histologic analysis of wound infection has been investigated. In the wound infection group after 24 h, the inflammatory cells, such as lymphocytes and granulocytes can be observed (Figs. 6d and S26), confirming the bacteria-related inflammation induced by the S. aureus and E. coli. Meanwhile, the number of inflammatory cells in the positive control group and the experimental groups at 14th day decrease and the epithelium and neovascularization can be observed in the wounds treated with CDGA. As well known, the collagen deposition is important during the wound healing process^50^. In the experimental group and the positive control group, the masson stainings of wound show larger areas of collagen fibrosis and the elastic fibers are arranged in a more orderly manner, interwoven into a net and dyed blue-violet. In addition, the weights of the mice in the experimental group and the positive control group increase as similar as the control group (Fig. 6e), confirming the low toxicity of CDGA. Thus, summarizing all these results, it can be revealed that the CDGA can efficiently kill bacteria, eliminate the inflammation of the affected area, and further safely promote the wound healing.

## Discussion

In summary, we have designed one kind of CDGA using a chemiexcited photosensitive CDs. With the efficient NIR CL emission and distinctive PDT capacity of CDs, the CDGA possess CL emission related to the levels of environmental H_2_O_2_ and offers high detection sensitivity, high specificity, superior imaging quality, and satisfactory safety for in vitro and in vivo bioimaging of bacteria-related inflammatory sites. Moreover, the ROS as these active substances can induce bacteria death effectively, endowing the CDGA as effective broad-spectrum antibacterial medicines by expressing the high ROS levels in wound infection. Considering the multiple advantages of simultaneous diagnosis and treatment, these findings open up new possibilities for convenient and effective curing and diagnosing of bacterial infection via a CL dynamic/guided therapeutic agent.

## Materials and methods

### Chemicals and materials

All the chemicals and reagents were purchased from Sigma-Aldrich and used without further purification. The fresh leaves were picked from the platanus, heather, pittosporum, bamboo, laurel planted in Zhengzhou University and cultivated for 3 days. Scallion leaves and chive leaves purchased from the supermarket.

### Preparation of the CDs

The CDs were obtained from the leaves of platanus with the solvothermal treatment. Actually, the fresh leaves of platanus heather, pittosporum, bamboo, laurel, scallion and chive were collected and dried at 60 °C to remove the water. Then, 1.0 g dried leaves and 20 ml ethanol solution ware transferred to a poly (tetrafluoroethylene) Teflon-lined autoclave (30 ml) and heated in an oven at 140 °C for 4 h. Subsequently, the reactor was cooled to room temperature. The resulting solution was obtained from supernatant solution through 0.22 µm polyethersulfone membrane to remove large particles. Then the crude products were purified via dry silica column chromatography using a mixture of ethyl acetate and petroleum ether as the eluent. After collecting black powder by removing the solvent under reduced pressure, the CDs were obtained for further characterization.

### Characterization

The surface morphology was characterized by a transmission electron microscope (TEM, JEOL JSM-IT100), an AIST-NT Smart atomic force microscope (AFM) and JSM-6700F scanning electron microscope (SEM). The X-ray diffractometer (Smartlab) using Cu k_α_ as the irradiation source was used to obtain the XRD patterns. The Fourier transform infrared spectra (FT-IR) were recorded on a Thermo Scientific Nicolet IS10 FTIR spectrometer. The X-ray photoelectron spectroscopy (XPS) was measured on a Kratos AXIS HIS 165 spectrometer with a monochromatized Al KR X-ray source (1486.7 eV). The fluorescence and chemiluminescence was measured by a F-7000 spectrofluorometer (Hitachi, Japan). The absorption spectrum was measured by an UV/VIS spectrophotometer (Hitachi, UH-4150). The fluorescence lifetime of the CDs solution was measured by Horiba FL-322 using a 370 nm Nano-LED monitoring the emission at 675 nm. The ESR spectra of the CDs were recorded by an electron paramagnetic resonance spectrometer (Bruker A300). The Dynamic light scattering (DLS) distribution was measured with Zetasizer Nano. The femtosecond transient absorption (TA) measurements were performed on a Helios pump-probe system (Ultrafast systems). The optical photograph was obtained from a realme V11 5G mobile phone (realme RMX3122, China).

### Synthesis of the CDGA

For perpetrating the CDGA, the CDs were dissolved in trichloromethane for 4 mg ml^−1^ by ultrasonic, the pluronic F-127 (50 mg ml^−1^) and bis[2,4,5-trichloro-6-carbopentoxyphenyl] oxalate (CPPO, 5 mg ml^−1^) were dissolved in trichloromethane by vortexing. Then, 0.4 mg CDs (4 mg ml^−1^), 50 mg F-127 (50 mg ml^−1^) and 5 mg CPPO (5 mg ml^−1^) were homogeneously mixed in flask. After the solvent was removed by a vacuum pump, then the dried mixture was dissolved with 2 ml Milli-Q water to make the concentration of CDGA was 200 μg ml^−1^.

### In vitro bioimaging of wound inflammatory infection

The mouse model of full-thickness skin wound (dorsum of the mouse) was used to image endogenous H_2_O_2_. Briefly, the wound of laboratory mice (*n* = 3) were dripped with 200 μl ZA (2 mg ml^−1^, Z4250, Sigma-Aldrich) for 48 h. The control mice were treated only using 200 μl saline. After 48 h treatment with the ZA, anesthetized mice (2% isoflurane in oxygen) were treated with 500 μl CDGA (200 μg ml^−1^ based on the CDs). Then, the mice were immediately put into the IVIS imaging chamber to acquire the luminescent signals with an exposure time of 3 min at every 5 min with open filter at 675 nm. The phosphate buffer saline (PBS) was used in control group.

### In vivo bioimaging of intraperitoneal inflammatory infection

The mouse model of peritonitis was used to image endogenous H_2_O_2_. Briefly, the C57BL/6J mice were injected intraperitoneally with 200 μl ZA (2 mg ml^−1^, Z4250, Sigma-Aldrich) for 48 h. For the inhibitor study, mice were treated 200 μl GSH (200 mg kg^−1^) for 24 h by intraperitoneally after ZA treatment. The control mice were treated only using 200 μl saline. After 48 h of the ZA treatment, anesthetized mice (2% isoflurane in oxygen) were treated with intraperitoneal injection of 200 μl CDGA (200 μg ml^−1^ based on CDs). Then, the mice were immediately put into the IVIS imaging chamber (Xenogen, Alameda, CA) to acquire luminescent signals with an exposure time of 3 min at every 5 min with open filter at 675 nm. The phosphate buffer saline (PBS) was used in control group.

### Antibacterial activity measurement

Five kinds of bacteria (S. aureus, P. aeruginosa, E. coli, S. mutans, and MRSA) were used to assess the antibacterial activity of CDGA by plate method. Briefly, the liquid of bacteria were diluted to 10^6^ CFU ml^−1^ with Luria-Bertani (LB) liquid medium. Then 1 ml different bacteria solution were added to the centrifuge tube with and without adding samples, and then incubated for 18 h in a incubator at 37 °C in dark. After the end of co-culture, the aseptic PBS was continuously diluted by ten times gradient (10^4^, 10^5^, 10^6^ times), and 100 μl was coated on LB solid culture medium. Finally, the samples were recorded.

### Bacteria staining and imaging

Two 12 ml bacterial culture tubes were added into 3 ml LB liquid medium, and a single colony from E. coli solid medium was added to the liquid culture medium. After incubated in a constant temperature oscillator (37 °C, 200 rpm) for 15 h, the bacterial liquid was diluted to 108 CFU ml^−1^ with LB liquid medium. Then, the 1 ml diluted bacteria suspension was added to the bacterial culture tube and the sterilized samples were added in the experimental group. After incubated at 37 °C for 24 h, the bacterial precipitates were collected after 8000 rpm centrifugation for 3 min. The bacterial precipitate was washed twice with PBS solution, and the dyeing solution was prepared (1 μl SYTO 9 and 2 μl PI were added to 1 ml PBS solution). Each 500 μl staining solution was used to suspend the bacteria. After 15 min stained at 37 °C without light, the test surface of the sample was recorded on a laser confocal microscope.

### Antibacterial capability of the CDGA

All animal experimental procedures and protocols were conducted in accordance with Animal Research: Reporting of In Vivo Experiments and the Guidelines of Intentional Standards of Animals Care of the Zhengzhou University (No. U2004168). A total of 12 laboratory mice were randomly divided into three groups of control group (treated with sterilized saline), positive control group (treated with adhesive bandage), and experimental group (treated with CDGA). After the laboratory mice were anesthetized with 10% chloral hydrate solution (3 ml kg^−1^) by intraperitoneal injection, the dorsal hairs of the laboratory mice were shaved with a hair clipper and a nummular full-thickness skin was resected with a scalpel. The diameter of the wound was about 2 cm and the operations were performed under aseptic conditions before the infection with the bacterial suspension contained S. aureus and E. coli, both of which had concentrations of 1.5 × 10^8^ CFU ml^−1^. Then, all the experimental laboratory mice were separately raised in cages at a standardized temperature and the wound dressings were changed once a day in each group in the period of 14 days. On the 3rd, 5th, 8th, 10th and 14th day from the start of treatment, the infected wounds were recorded. On the 14th day, all the experimental mice were sacrificed by euthanasia. Subsequently, the tissue collected from the infected wound was fixed in 4% paraformaldehyde, embedded in paraffin, and cut into 4 μm sections. The samples were stained with hematoxylin-eosin (H&E) and Masson and observed with a brightfield microscope. Within 14 days treatment, the weights of mice in each group were recorded, and the weight change curve was plotted. The wound areas were calculated by the software of ImageJ.

## Supplementary information

Supplementary Information

## Data Availability

All the data and methods needed to evaluate the conclusions of this work are presented in the main text. The data that support the findings of this study are available from the corresponding author upon request.
