# Supplementary material for: Chemiluminescent carbon nanodots for dynamic and guided antibacteria
Source: Light Sci Appl. 2023 May 4;12:104. doi: 10.1038/s41377-023-01149-8 (PMC10160024; doi:10.1038/s41377-023-01149-8)
Supplement: Supplementary file 1 — Supplementary Information [file 41377_2023_1149_MOESM1_ESM.docx]

Supplementary Information for

**Chemiluminescent carbon nanodots for dynamic and guided antibacteria**

Jiang-Fan Han^1^, Qing Lou^1,*^, Zhong-Zheng Ding^1^, Guang-Song Zheng^1^, Qing-Chao Ni^1^, Run-Wei Song^1^, Kai-Kai Liu^1^, Jin-Hao Zang^1^, Lin Dong^1^, Cheng-Long Shen^1,*^, Chong-Xin Shan^1,^^[[1]](#footnote-1)^*

*^1^Henan Key Laboratory of Diamond Optoelectronic Materials and Devices, Key Laboratory of Material Physics, Ministry of Education, School of Physics and Microelectronics, Zhengzhou University, Zhengzhou 450052, China.*

**Table of Content**

1. **Fig. S1.** Schematic illustration of the preparation of CDs.
2. **Fig. S2.** The CDs prepared from different conditions.
3. **Fig. S3.** The productivity of CDs.
4. **Fig. S4.** The fluorescence spectra of CDs prepared from different plant leaves.
5. **Fig. S5.** The atomic force microscope (AFM) image of the CDs (inset: the height profile along the line).
6. **Fig. S6.** The size distribution of the CDs in aqueous solution.
7. **Fig. S7.** The stability of the chemiluminescent CDs.
8. **Fig. S8.** Excitation-emission matrices of the CDs in different solvents.
9. **Fig. S9.** Photoluminescence (PL) emission spectra and time-resolved decay spectra of the CDs in different concentrations of ethanol solution.
10. **Fig. S10.** The photoluminescence (PL) emission spectra of the CDs with different excitation wavelengths in different solvents.
11. **Fig. S11.** UV–vis absorption spectra of CDs in different solvents.
12. **Fig. S12.** Chemiluminescence (CL) characterization of lucigenin and CDs.
13. **Fig. S13.** The size distribution of the CDGA.
14. **Fig. S14.** The SEM image and the optical photograph of the CDGA film.
15. **Fig. S15.** The photoluminescence (PL) excitation (EX) and emission (EM) spectra of the CDGA and UV–vis absorption spectra of the F-127, F-127 + CPPO and CDGA in H_2_O solution.
16. **Fig. S16.** Chemiluminescence (CL) and photoluminescence (PL) spectra of the CDGA.
17. **Fig. S17.** The CL response of the CGDA to various reactive oxygen species (ROS) in the CL analysis instrument.
18. **Fig. S18.** The CL emission of the CDGA recorded by the IVIS system.
19. **Fig. S19.** The CL images of mice treated with and without the ZA in superficial wound.
20. **Fig. S20.** The tissue penetration depth of CD-based chemiluminescence and fluorescence with CDs.
21. **Fig. S21.** The stability of the CDGA film.
22. **Fig. S22.** The influence factors about the CDGA-based CL image.
23. **Fig. S23.** The flat counting images of E. coli treated with the F-127, F-127 + CDs, F-127 + CPPO and CDGA under dark condition.
24. **Fig. S24.** The antibacterial activity of different concentrations of CDGA.
25. **Fig. S25.** The optical photograph of the pork wounds covered with CDGA after washed by saline.
26. **Fig. S26.** Images of H&E staining.


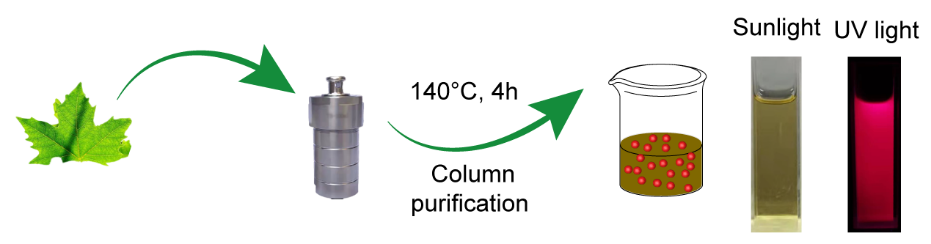


**Fig. S1.** Schematic illustration of the preparation of CDs.


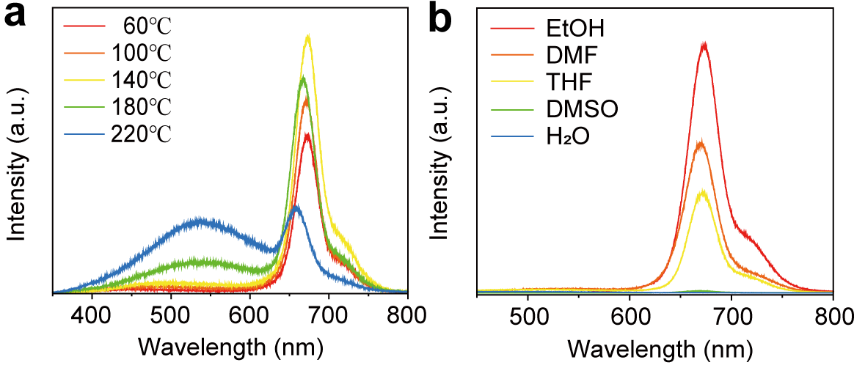


**Fig. S2. a** The PL spectra of the products obtained from 60 ℃ to 220 ℃. **b** The PL spectra of the products obtained in EtOH, DMF, THF, DMSO and H_2_O.


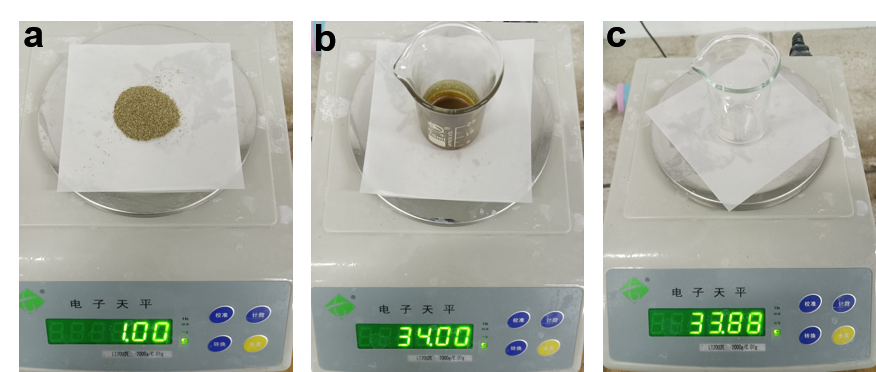


**Fig. S3.** The productivity of CDs. **a** The mass (M) of the platanus leaves. **b** The total mass (m_1_) of CDs and beaker. **c** The mass (m_2_) of beaker.

**Note:** The productivity (Q) of the CDs was determined according to the following equation:

$$Q=\frac{m}{M}\times100\%$$

where m and M are the mass of the as-prepared CDs and total mass of precursor.


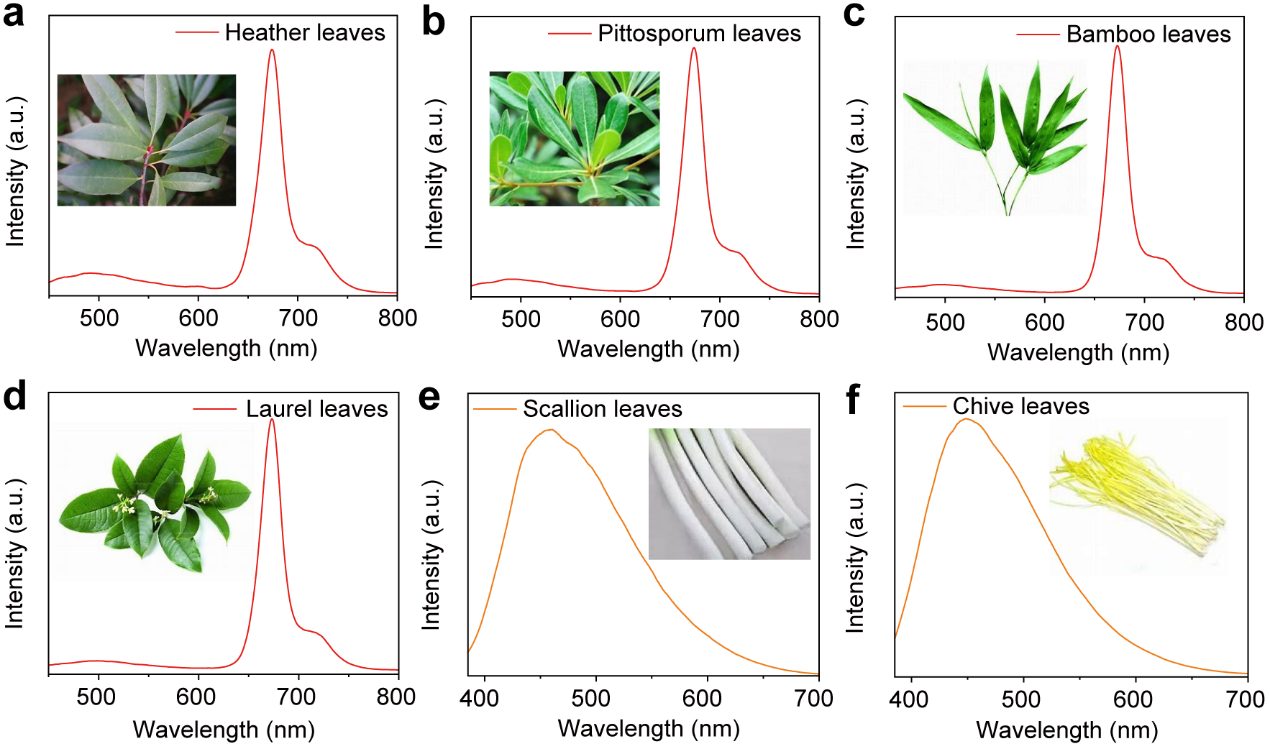


**Fig. S4.** **a-f** The fluorescence spectra of the products prepared from the plant leaves of heather leaves **a**, pittosporum leaves **b**, bamboo leaves **c**, laurel leaves **d**, scallion leaves **e**, and chive leaves **f**.


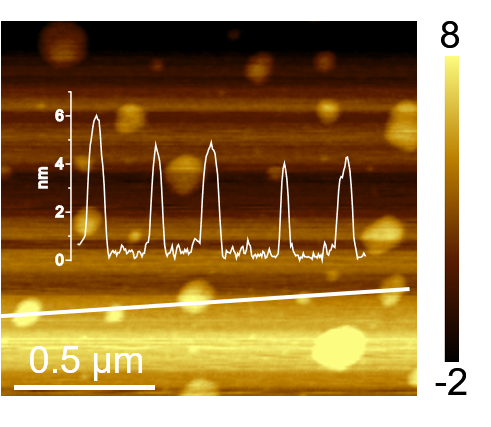


**Fig. S5.** The atomic force microscopy (AFM) image of the CDs (inset: the height profile along the line).


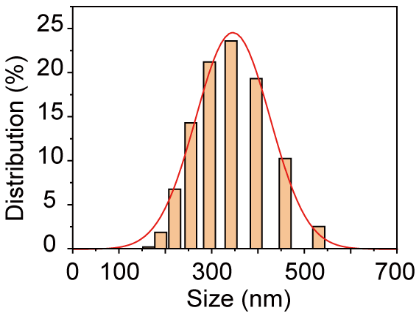


**Fig. S6.** The size distribution of the CDs in aqueous solution.


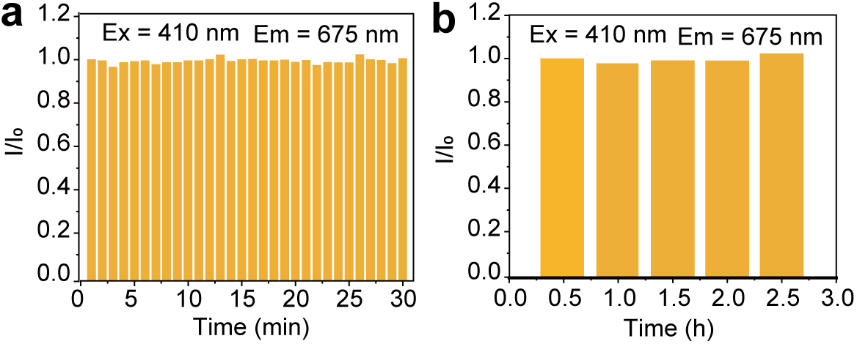


**Fig. S7.** **a** The PL intensity of CDs after irradiated with a 365 nm UV lamp. **b** The PL intensity of the CDs after exposed to natural light.


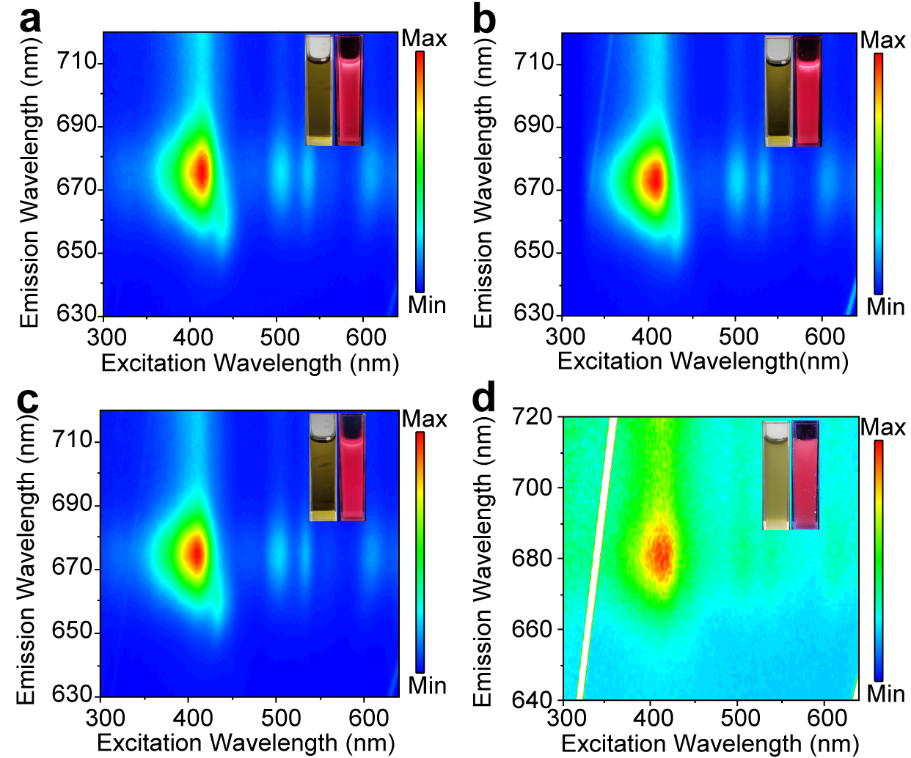


**Fig. S8.** **a-d** Excitation-emission matrices of the CDs in DMSO solution **a**, DMF solution **b**, THF solution **c** and H_2_O solution **d**. (inset: the fluorescence image of the CDs under sunlight (left) and 365 nm UV excitation (right)).


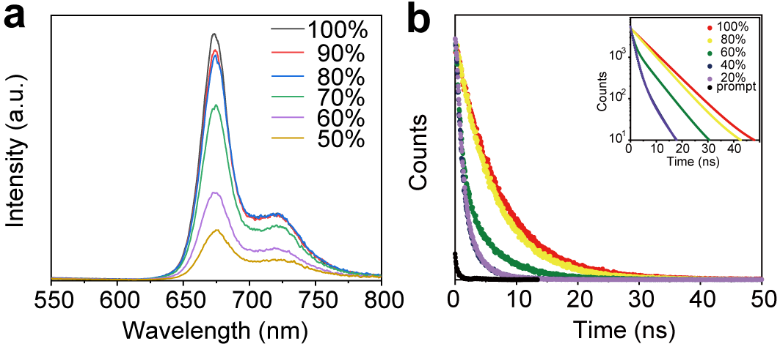


**Fig. S9.** **a** The photoluminescence (PL) emission spectra of the CDs in different concentrations of ethanol solution. **b** Time-resolved decay spectra of the CDs in different concentrations of ethanol solution.


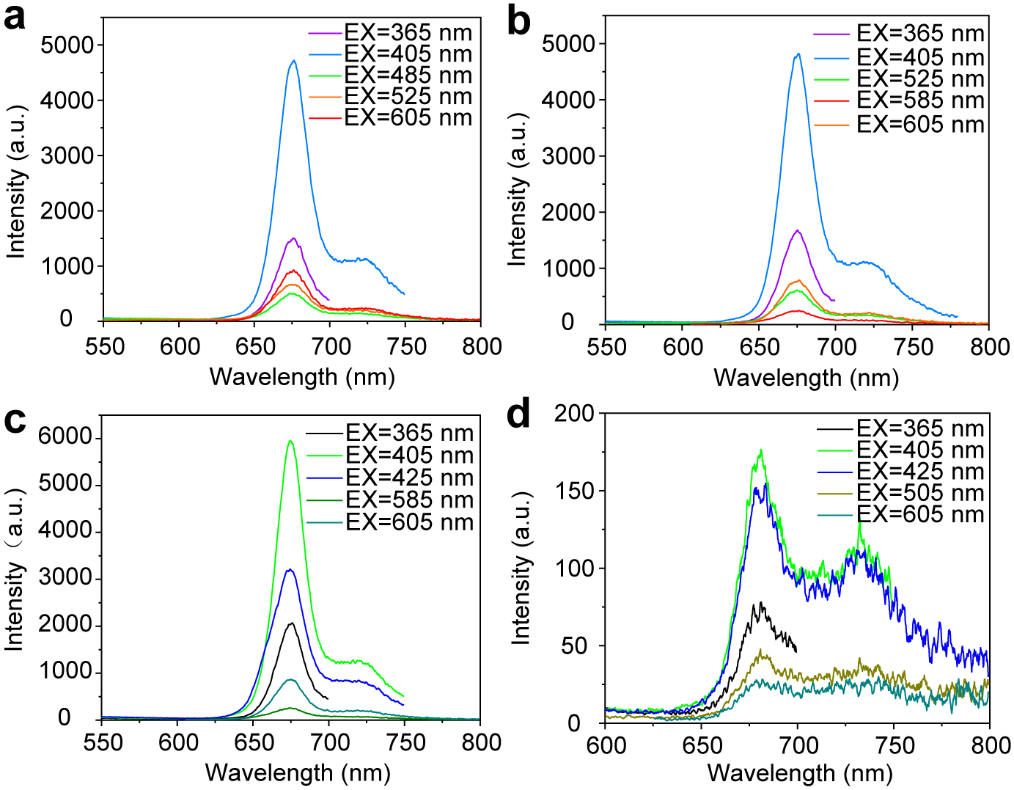


**Fig. S10. a-d** The photoluminescence (PL) emission spectra of the CDs with different excitation wavelengths in DMSO solution **a**, DMF solution **b**, THF solution **c** and H_2_O solution **d**.


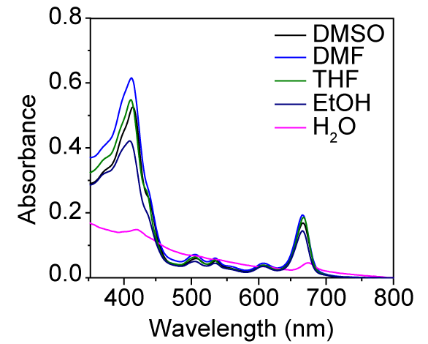


**Fig. S11.** The UV-vis absorption spectra of CDs in different solvents.


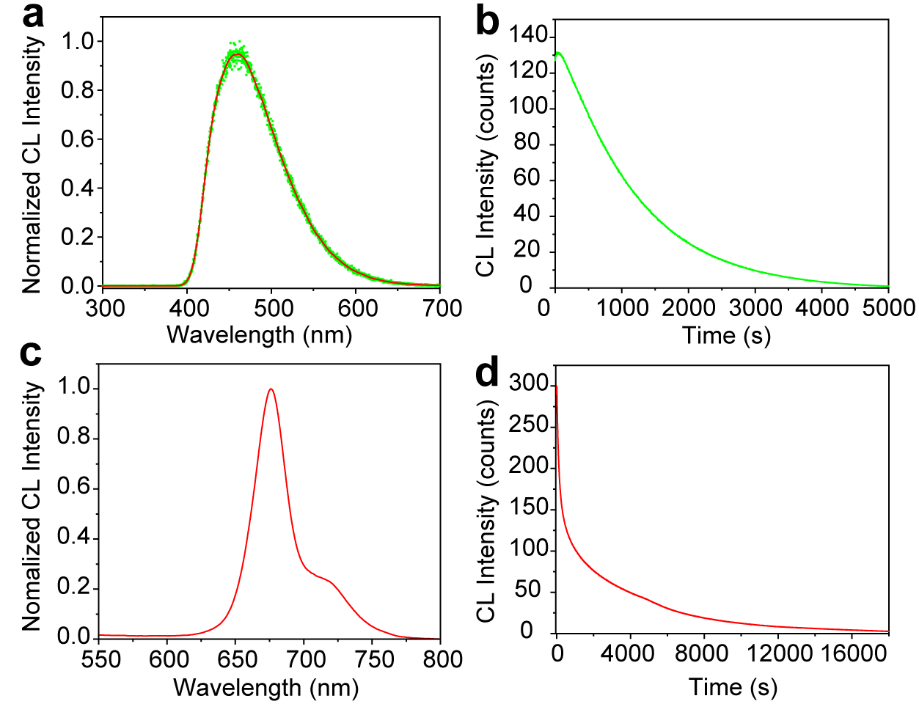


**Fig. S12. a-b** The CL spectrum of lucigenin-H_2_O_2_ **a** and the CL intensity decay curves at 475 nm after adding H_2_O_2_ into the lucigenin solution **b**, (EM slit = 5 nm, PMT voltage = 950 V). **c** The CL spectrum of CDs. **d** The CL intensity decay curves at 675 nm after adding H_2_O_2_ into the CPPO and CDs.

**Note:** The CL QYs of the CDs were measured using lucigenin as a reference with a known QY of 11.3 × 10^−3^ einsteins mol^−1^ at pH = 13.8 according to our previous reports. With the CL spectra and kinetic curves, the CL QYs were calculated according to the following equations:

$\varphi_{CL}=\frac{\text{Q}\times f_{luc}\times f_{photo}}{n_{\text{CPPO}}}(einsteins/mol)$ (1)

$f_{luc}=\frac{\varphi_{luc}\times n_{luc}}{Q_{luc}}$ (2)

$f_{photo}=\frac{f(\lambda_{s})}{f(\lambda_{luc})}$ (3)

Where *Փ* is the CL QY of the CDs, *Q* is the total light emission obtained by integration of emission intensity under time curves. In the equation, *f_luc_* is obtained by measuring the emission kinetics of lucigenin reaction performed in standard conditions (λ = 475 nm). *f_photo_* is obtained from the sensitivity of CL spectrum normalized at the emission wavelength (λ = 475 nm) of the lucigenin standard, *f* (*λ_luc_*), and at the emission wavelength (λ = 675 nm) of the CPDs or CNGs, *f* (*λ_s_*). n is the number of moles of lucigenin (n_luc_) or the number of moles of CPPO (n_cppo_).


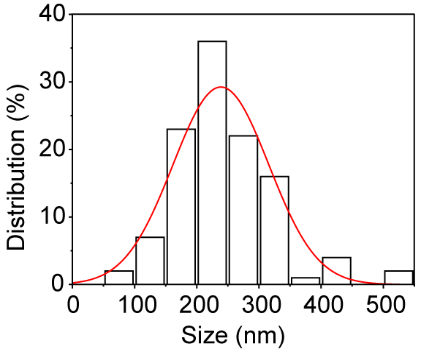


**Fig. S13.** The size distribution of the CDGA.


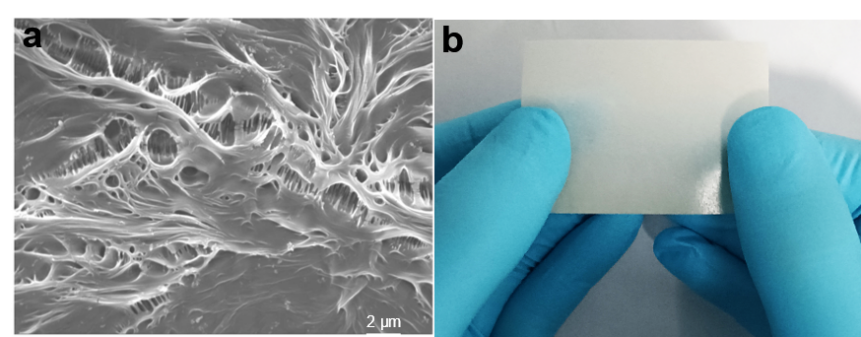


**Fig. S14.** **a** The SEM image of the CDGA film. **b** The optical photograph of the CDGA film.


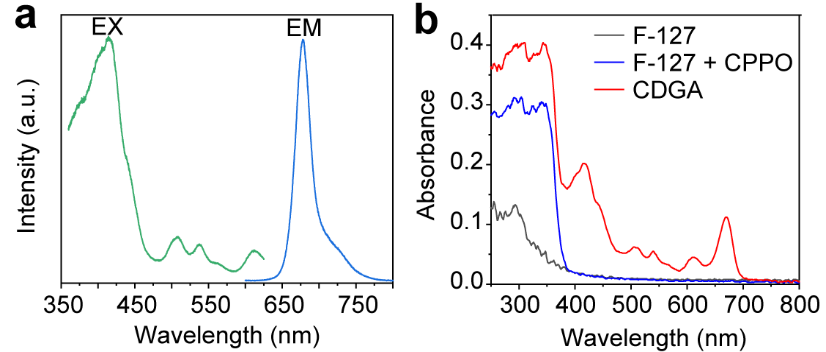


**Fig. S15.** **a** The photoluminescence (PL) excitation (EX) and emission (EM) spectra of the CDGA in H_2_O solution. **b** UV–vis absorption spectra of the F-127, F-127 + CPPO and CDGA in H_2_O solution.


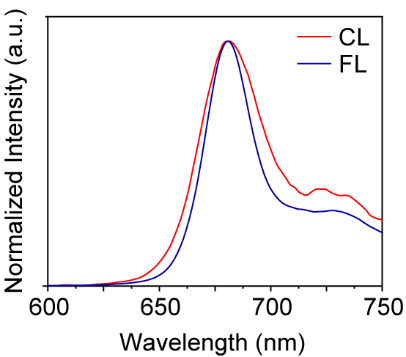


**Fig. S16.** Chemiluminescence (CL) and photoluminescence (PL) spectra of the CDGA.


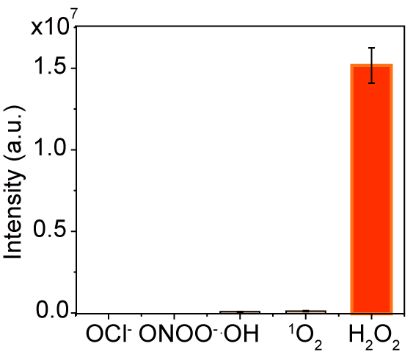


**Fig. S17.** The CL response of the CGDA to various reactive oxygen species (ROS) in the CL analysis instrument.

**Note:** Thereinto, OCl^−^ solution was prepared by directly diluting commercially available NaOCl. •OH was generated by reacting Fe^2+^ with H_2_O_2_. ONOO^−^ stock solution was prepared by directly diluting commercially available NaONOO. ^1^O_2_ was produced from the H_2_O_2_-molybdate ions (Na_2_MoO_4_) system.


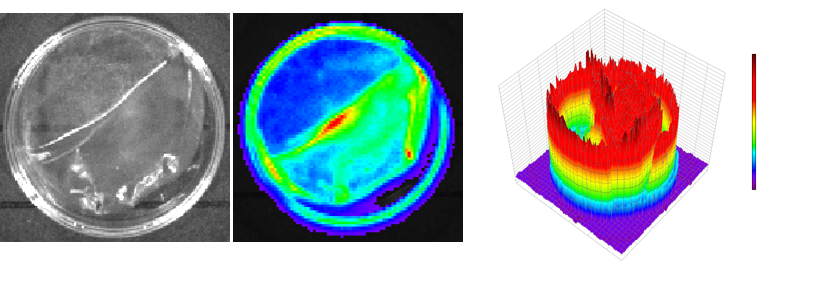


**Fig. S18.** The CL emission of the CDGA recorded by the IVIS system.


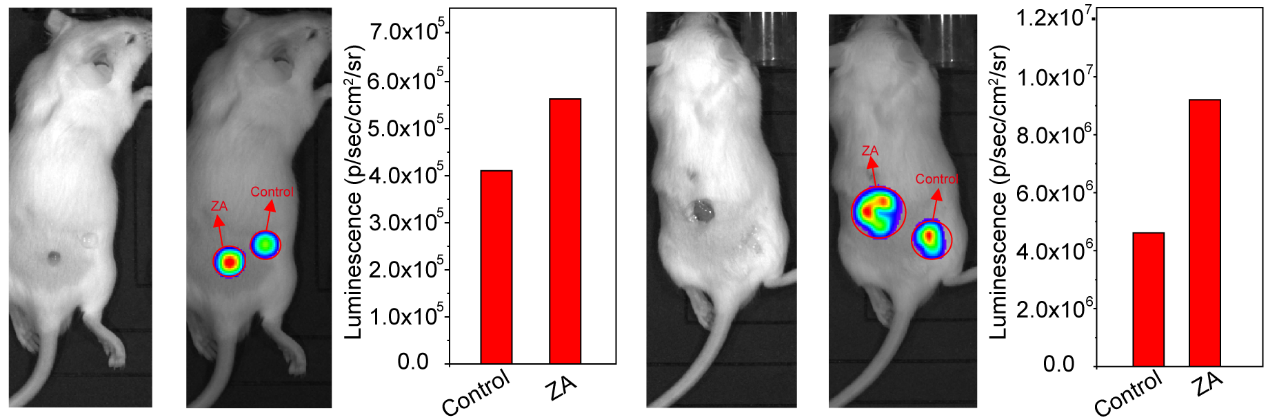


**Fig. S19.** The CL images of mice treated with and without the ZA in superficial wound.


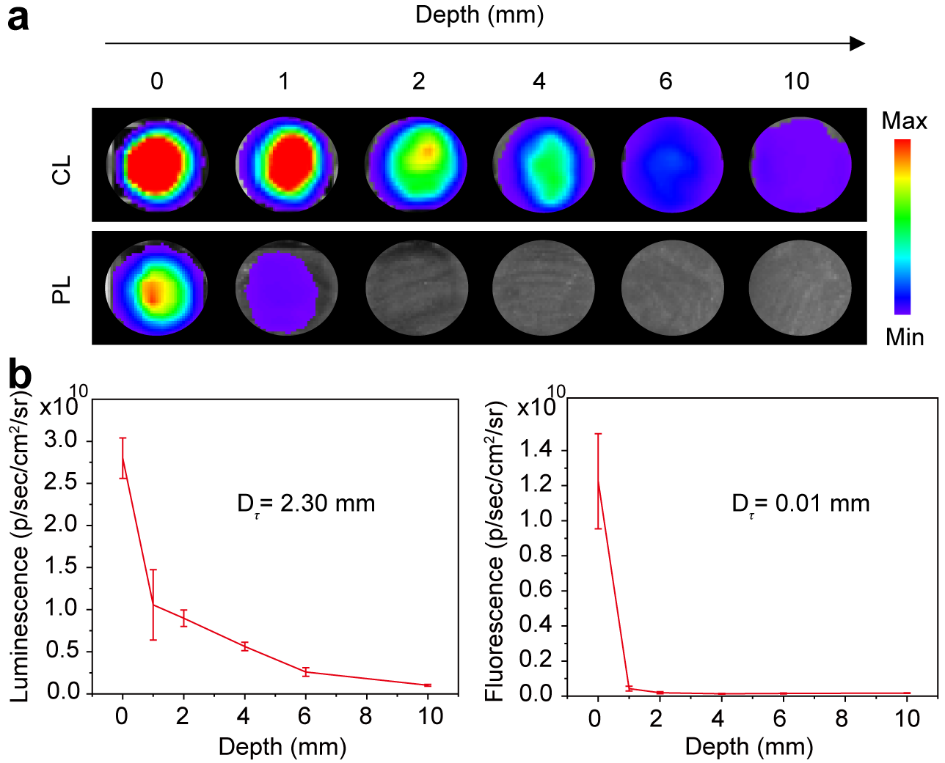


**Fig. S20. a** The CL of the CDGA and the FL image of the CDs through stacked slices of chicken at different depth. **b** The corresponding CL intensity of CDGA and FL intensity of the CDs (D*_τ_* is the effective tissue penetration depth).


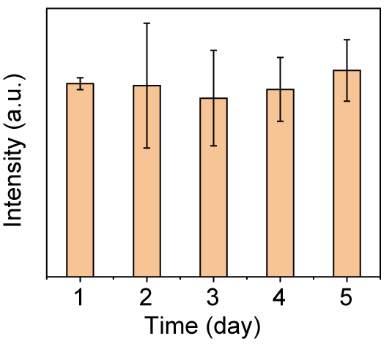


**Fig. S21.** The CL intensity of the CDGA film at 675 nm after stored for different time.


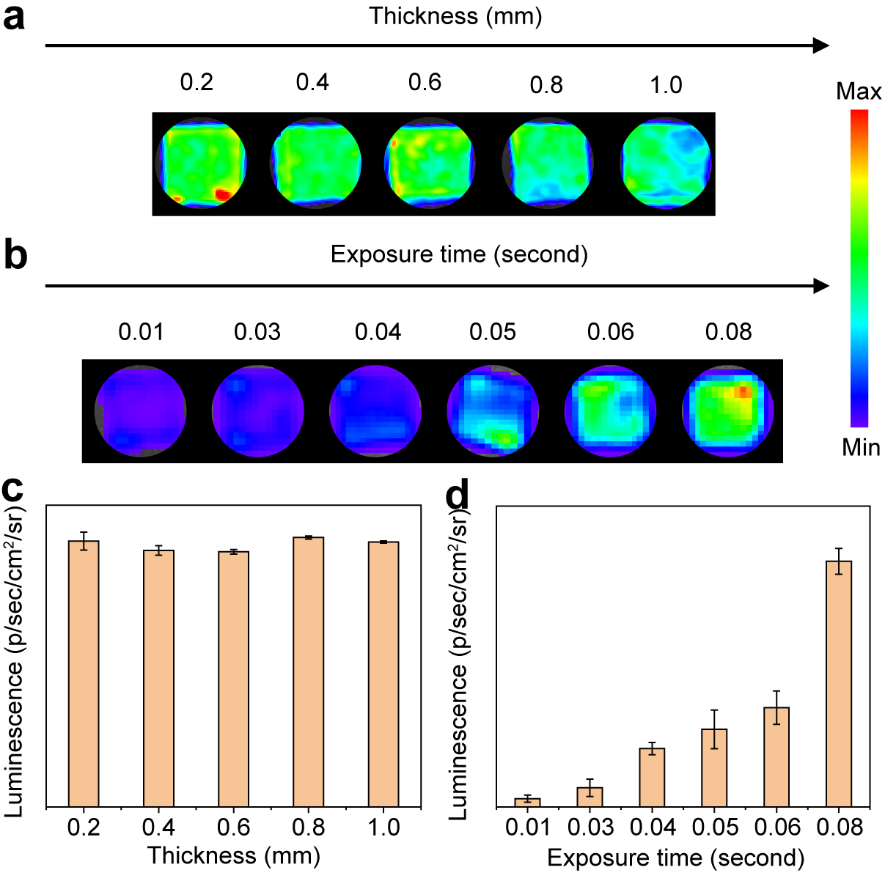


**Fig. S22.** **a** The CL image of the CDGA films with different thickness at 675 nm. **b** The CL image of the CDGA films with different exposure time. **c** The CL intensity of the CDGA films with different thickness at 675 nm. **d** The CL intensity of the CDGA films with different exposure time.


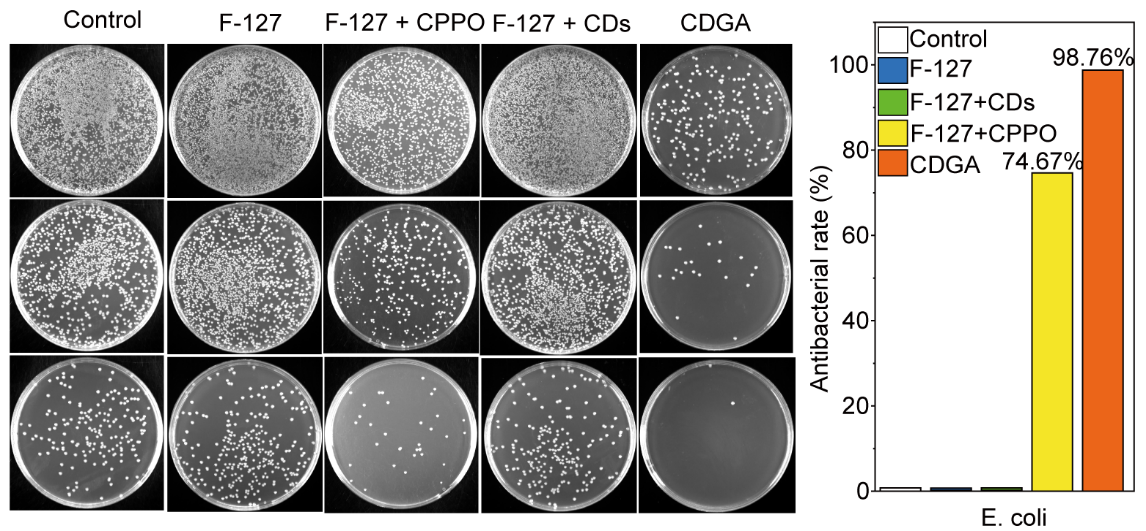


**Fig. S23.** The flat counting images of E. coli treated with the F-127, F-127 + CDs, F-127 + CPPO and CDGA under dark condition.


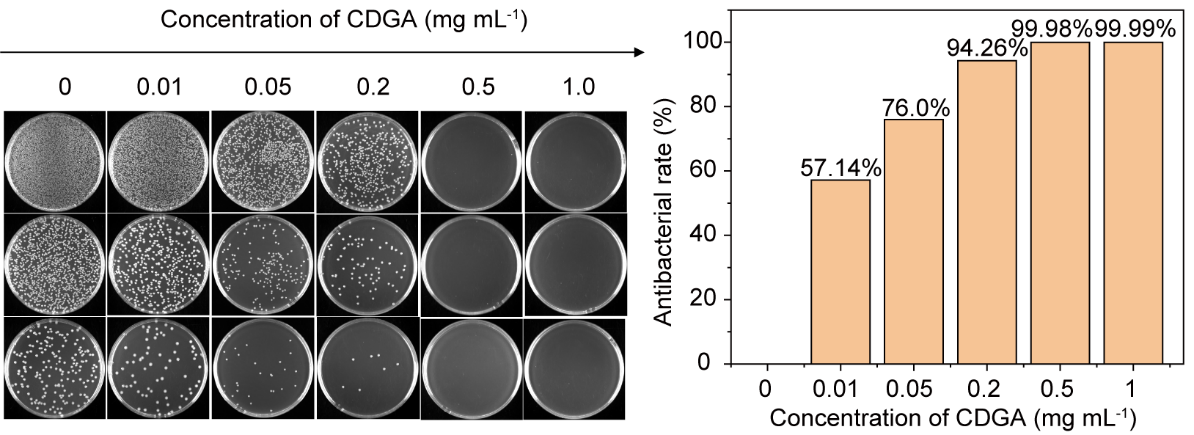


**Fig. S24.** **a** The flat counting images of E. coli treated with the different concentrations of CDGA under dark condition. **b** The antibacterial rate of the different concentrations of CDGA.


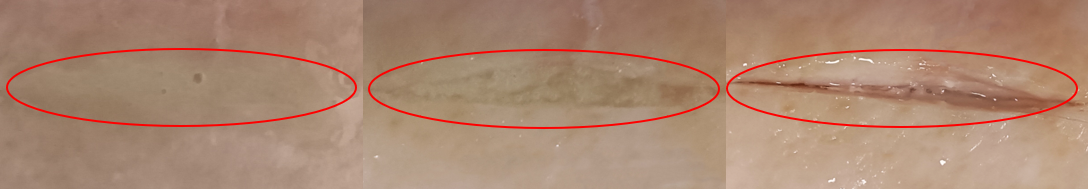


**Fig. S25.** The optical photograph of the pork wounds covered with CDGA after washed by saline.


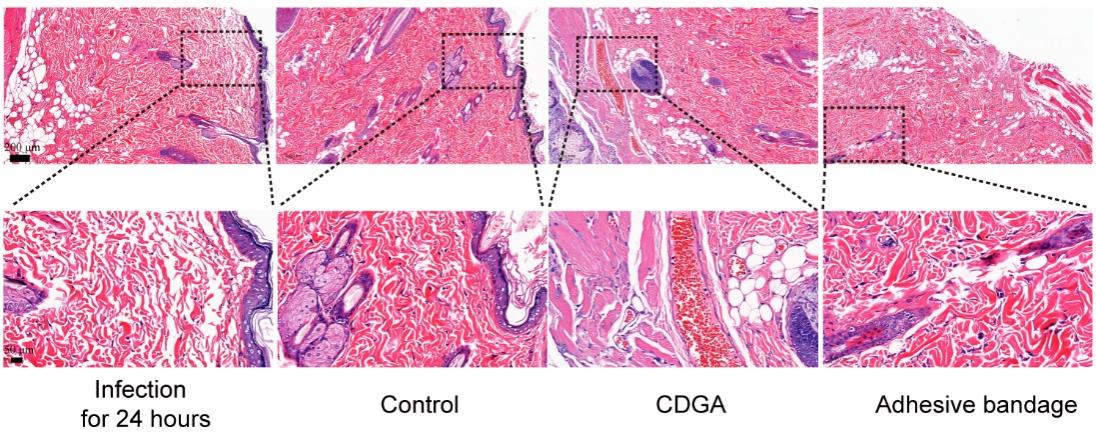


**Fig. S26.** Images of H&E staining, including infection for 24 h and treatment for 14 days. The scale bars are 200 μm and 50 μm.

1. * Correspondence: louqing1986@zzu.edu.cn (Q.L.), phyclshen@zzu.edu.cn (C.L.S.), cxshan@zzu.edu.cn (C.X.S.) [↑](#footnote-ref-1)
